# Multi-and many-objective optimization: present and future in *de novo* drug design

**DOI:** 10.3389/fchem.2023.1288626

**Published:** 2023-12-18

**Authors:** Jaqueline S. Angelo, Isabella A. Guedes, Helio J. C. Barbosa, Laurent E. Dardenne

**Affiliations:** Coordenação de Modelagem Computacional, Laboratório Nacional de Computação Científica, Petrópolis, Brazil

**Keywords:** drug discovery, *de novo* drug design, evolutionary algorithms, multi-objective optimization, many-objective optimization

## Abstract

*de novo* Drug Design (dnDD) aims to create new molecules that satisfy multiple conflicting objectives. Since several desired properties can be considered in the optimization process, dnDD is naturally categorized as a many-objective optimization problem (ManyOOP), where more than three objectives must be simultaneously optimized. However, a large number of objectives typically pose several challenges that affect the choice and the design of optimization methodologies. Herein, we cover the application of multi- and many-objective optimization methods, particularly those based on Evolutionary Computation and Machine Learning techniques, to enlighten their potential application in dnDD. Additionally, we comprehensively analyze how molecular properties used in the optimization process are applied as either objectives or constraints to the problem. Finally, we discuss future research in many-objective optimization for dnDD, highlighting two important possible impacts: i) its integration with the development of multi-target approaches to accelerate the discovery of innovative and more efficacious drug therapies and ii) its role as a catalyst for new developments in more fundamental and general methodological frameworks in the field.

## 1 Introduction

Life involves choices, reaching decisions, and seeking compromises. The major challenge lies in managing the conflict between the various goals and objectives Miettinen (1999). As in many real-world problems, the discovery of a new drug with desired pharmacological and pharmacokinetic properties has several objectives to be considered. For instance, in the search for new therapeutic drugs, the maximization of (i) the potency of the drug, (ii) the structural novelty, (iii) pharmacokinetic profile, the minimization of (iv) synthesis costs, and (v) unwanted side effects are desired goals to be optimized Rosenthal and Borschbach (2017); Lambrinidis and Tsantili-Kakoulidou (2021). Thus, designing new effective and safe drugs is inherently a problem with diverse objectives to be optimized concurrently.

Many computational tools have been developed to assist in the design of novel drug-like molecules, such as quantitative structure-activity relationship (QSAR) Jana et al. (2020); Wang Z. et al. (2021); Socha et al. (2023), molecular docking and affinity prediction through machine learning-based scoring functions Guedes et al. (2014) Santos et al. (2020); Guedes et al. (2021). Flurbiprofen, vaborbactam, and atazanavir are commercially approved drugs discovered by computer-aided drug design Sabe et al. (2021). The term “*de novo*” in Latin means “anew”, “afresh”, or “from the beginning”. The goal of *de novo* drug design (dnDD) is to create novel molecules with desirable properties from scratch. In this context, multiple properties mean multiple objectives to be optimized.

The problem of optimization refers to the task of discovering feasible solutions until no better solution can be found. The quality of a solution is evaluated based on an objective, while the feasible region represents a set of conditions or constraints that limit the solutions to the problem. In dnDD, an objective can be expressed by various properties of interest, such as a similarity score to a known ligand or a binding score with a target receptor Nicolaou et al. (2009). At the same time, the constraints may be any useful function, such as chemical stability and synthetic feasibility Nicolaou et al. (2012).

For a long time, researchers in dnDD neglected the presence of multiple conflicting objectives Nicolaou et al. (2009), such as simultaneously maximizing the potency of a drug to a specific target and minimizing known side effects, which are naturally present in this type of problem. Due to the complexity of designing a new molecule, a single objective may not cover the multi-faceted design issues. Thus, researchers seek new techniques or design strategies that simultaneously consider the multiple aspects of this class of problems.

While in a single-objective optimization problem (SingleOOP), the goal is to optimize only one objective function, in a multi-objective optimization problem (MultiOOP), more than one objective must be simultaneously optimized. If the objective functions are not conflicting, a solution can be found where each objective reaches its optimum value. However, in MultiOOPs, those objectives are frequently conflicting (*i.e.*, the improvement of one objective leads to the degradation of another objective), and also non-commensurable (*i.e.*, when dealing with objectives that have different units or scales of measurement). In this case, there is usually no optimal solution but a set of trade-off solutions representing a compromise between the conflicting objectives. Such solutions are called *non-dominated* solutions, which form the *Pareto (optimal) set*, which is the set of solutions that are all equally optimal concerning the considered objectives.

An automatic dnDD can accelerate the overall drug discovery process but can be complex and computationally demanding. Since LEGEND, the first dnDD technique proposed in 1991 Nishibata and Itai (1991), numerous other methods have been developed to assist researchers in drug discovery. In particular, Evolutionary Algorithms (EAs) have been widely used to find the optimal solution(s) in *de novo design* Devi et al. (2015); Le and Winkler (2015). EAs are population-based metaheuristics in which a collection of candidate solutions evolve under specified selection rules to a state that minimizes/maximizes a general cost function. In contrast to classical search methods that usually aggregate the objective functions into one objective, EAs can be easily applied to multi-objective optimization problems due to their population-based nature, allowing EAs to find a set of non-dominated solutions in a single run. Such methods are called multi-objective EAs (MultiOEAs).

Research in evolutionary computation has primarily focused on problems having two or three objectives Coello et al. (2007). However, many real-world problems have several (more than three) objectives in their formulation, *e.g.*, car side-impact Deb et al. (2009), mechanical engineering problems Ursem and Justesen (2012), water resource management Asafuddoula et al. (2015), routing planning in agricultural mobile robots Zhang et al. (2022), wireless sensor network deployment Ben Amor et al. (2022), among others. Particularly, dnDD has intrinsically various objectives to optimize, clearly more than three Rosenthal and Borschbach (2017); Lambrinidis and Tsantili-Kakoulidou (2021).

“Multi-objective” refers to scenarios involving three objective functions at most, while “many-objective” is usually adopted to specify problems with more than three objectives. The growing interest in the area of many-objective optimization (ManyOO) motivated the study and the design of new optimization techniques capable of solving problems with four to twenty or even more objective functions, as occurs in the nurse scheduling problem, with 25 objectives to be considered Burke et al. (2004); Sülflow et al. (2007). However, problems with many objectives present additional challenges compared to low-dimensional problems, *e.g.*, finding a good approximation of the Pareto set. Many-objective optimization in real-world applications has many decision design components that users commonly undertake Deb et al. (2023).

Over the past three decades, many-objective EAs (ManyOEAs) have been the subject of extensive research and practical implementation in various real-world applications, making them a widely studied and applied field Safi et al. (2018); Sato and Ishibuchi (2023).

This paper aims to review and enlighten the potential application of ManyOO in dnDD, with particular emphasis on EAs. There have been a limited number of examples where ManyOEAs are applied to dnDD and published in scientific literature. Thus, the main contributions of this paper are:

• introduce the definition of ManyOOPs and discuss the main challenges that impact the choice and the design of evolutionary techniques when the number of objectives of an optimization problem increases;
• review the different classifications of EAs for solving multi-objective and many-objective optimization problems;
• present and classify some existing MultiOEAs for dnDD;
• enumerate and categorize several ManyOEAs, from the most representative to the most recent state-of-the-art techniques, which have the potential to be applied in dnDD;
• present recent research that employs machine learning techniques, as they are emerging as a promising class of methods for multi-objective and possibly for many-objective dnDD;
• provide a comprehensive analysis of how the various molecular properties used in the optimization process are applied as either objectives or constraints to the problem.

**FIGURE 1:**
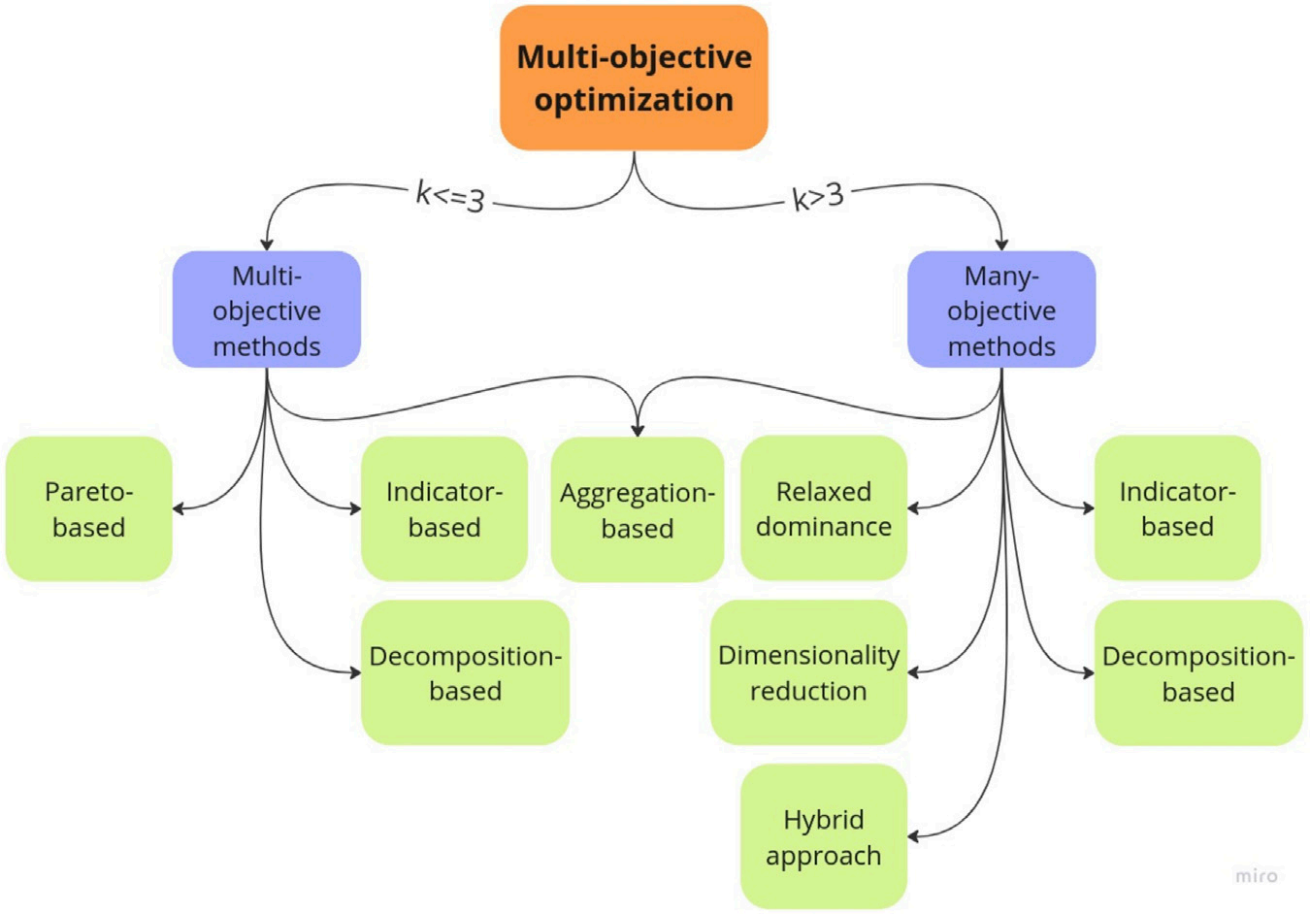
Classes of optimization methods adopted in this work, where *k* is the number of objectives. Flowchart generated with the Miro program (https://miro.com/).

As far as we know, this is the first review covering the application of ManyOO methodologies for the dnDD in addition to the MultiOO approaches. To organize and classify the different methodologies employed in these two main areas, “multi-objective” and “many-objective”, herein, we classify the optimization algorithms into two classes: *multi-objective methods*, in which problems are defined with two or three objectives, and *many-objective methods*, where problems contain four or more objectives, as illustrated in Figure 1.

The paper is organized as follows. Section 2 provides basic concepts and definitions regarding multi-objective optimization. Section 3 presents solutions methods for solving MultiOOPs, including classical techniques and MultiOEAs. Section 4 discuss the main challenges faced when dealing with ManyOOPs. A classification of ManyOEAs is provided, presenting recent and representative methods for each approach. In Section 5, we describe applications in dnDD, focusing on the two classifications of methods proposed (multi-objective and many-objective), presenting not only techniques based on EAs but also based on Machine Learning (ML) methods. Recently, hybrid approaches have emerged, combining EAs with ML techniques to increase the potential of these two classes of methods. Additionally, we will discuss how researchers distinguish objectives and constraints among the many molecules’ properties in dnDD. Perspectives concerning the ManyOO approach in dnDD are given in Section 6. The conclusions are presented in Section 7.

## 2 Key concepts in the multi-objective optimization problem

A MultiOOP can be written as

$$\begin{array}{cccc} \text{Minimize/Maximize} & & F\left(\text{x}\right)={\left(f_{1}\left(\text{x}\right),f_{2}\left(\text{x}\right),\ldots ,f_{k}\left(\text{x}\right)\right)}^{T} & \\ \text{subject to} & & g_{j}\left(\text{x}\right)\leq 0, & j=1,2,\ldots ,J;\\ & & h_{p}\left(\text{x}\right)=0, & p=1,2,\ldots ,P;\\ & & x_{i}^{l}\leq x_{i}\leq x_{i}^{u}, & i=1,2,\ldots ,n \end{array}$$ (1)

where we have *k* ( ≥ 2) objective functions $f_{i}:R^{n}\to R$ that must be minimised or maximized. For convenience, we will treat the MultiOOP as a minimization problem; however, if an objective function *f*_*k*_(**x**) needs to be maximized, then it is equivalent to minimizing the function −*f*_*k*_(**x**). The vector of objective functions is denoted by **F**(**x**), where $\text{x}={\left(x_{1},x_{2},\ldots ,x_{n}\right)}^{T}$ is a solution (decision) vector with *n* variables. Associated with the problem, there are *J* inequality and *P* equality constraints, such that $g:R^{n}\to R^{J}$ and $h:R^{n}\to R^{P}$ , respectively. The last set of constraints in (1) is the bound constraints that restrict each variable of the decision vector **x** to a value within a lower $x_{i}^{l}$ and an upper $x_{i}^{u}$ bound, with *i* = 1, …, *n*. A feasible solution is any solution **x** that satisfies all constraints, and the set of all feasible solutions constitutes the feasible (set) region $S\subset R^{n}$ , which is a subset of the *decision space*.

**FIGURE 2:**
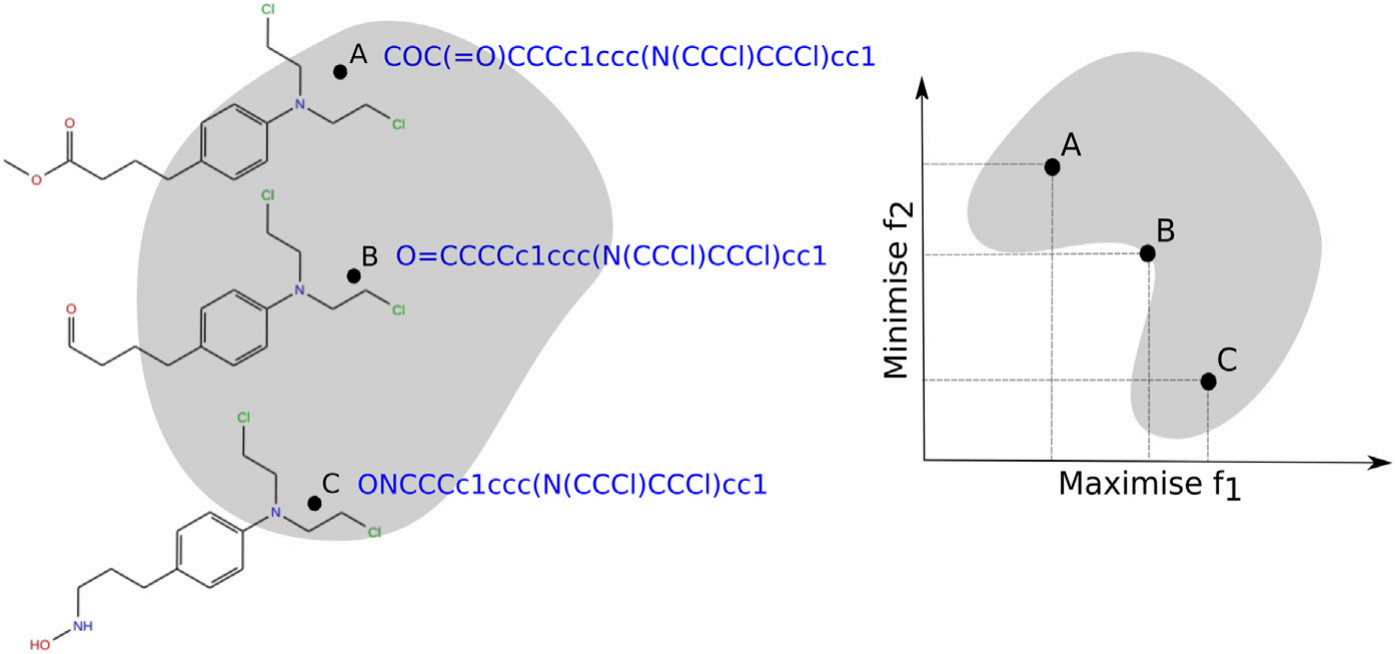
Hypothetical decision space (left) and objective space (right) associated with objectives *f*_1_ and *f*_2_ for the molecules (**A** **,** **B** **and** **C**) represented by the SMILES strings.

The notable difference between single-objective and multi-objective optimization is that in MultiOOP, the objective functions constitute a multi-dimensional space called the *objective space* $Z\subset R^{k}$ . The elements of *Z* correspond to the objective vector $\text{z}=F\left(\text{x}\right)={\left(z_{1},z_{2},\ldots ,z_{k}\right)}^{T}$ , where *z*_*i*_ = *f*_*i*_, *∀i* = 1, . . , *k*. For each solution vector **x** in the decision space, there is an objective vector **z** in the objective space. Such mapping relates an *n*-dimensional solution vector (in the decision space) to a *k*-dimensional objective vector (in the objective space). An illustrative example of these two spaces is shown in Figure 2. For clarity, suppose that *A*, *B*, and *C* are three different compounds and *f*_1_ and *f*_2_ are affinity predictions given in *pK*_*i*_ of each molecule against two different targets (*e.g.*, one therapeutic protein and one protein considered as an off-target related to side effects). Our objectives are to maximize the affinity value of these compounds against “target 1” and to minimize the affinity against “target 2”. Hence, for each solution in the decision space (*e.g.*, represented by the SMILES of a particular molecule), there is a corresponding point in the objective space (represented by the affinity prediction of the compound against each target).

### 2.1 Pareto optimality

In MultiOOP, several objectives must be simultaneously optimized. Usually, no single solution would give the best values for all the objective functions. Instead, in a typical MultiOOP with conflicting objectives, a set of solutions is superior to the others when all objectives are considered. Such solutions are those where none of the objectives can be improved without deteriorating at least one of the other objectives. Those solutions are called *Pareto-optimal solutions* due to the Italian economist Vilfredo Pareto, who introduced this theory in 1896 Pareto (1964). The optimal solution for a MultiOOP is based on the Pareto optimality concept, in which two solutions are compared based on whether one dominates the other.

Definition 2.1. *A decision vector* **x** ∈ *S* *dominates another vector* **y** ∈ *S* (**x** ≺**y**) *if and only if*

$$f_{i}\left(\text{x}\right)\leq f_{i}\left(\text{y}\right)\;\forall i\in \left\{1,\ldots ,k\right\}\;\mathrm{and}\;\exists j\in \left\{1,\ldots ,k\right\}:f_{j}\left(\text{x}\right)<f_{j}\left(\text{y}\right)$$

*where* *k* *is the number of objectives.*

Definition 2.2. *A decision vector* x* ∈ *S* *is a Pareto-optimal solution if no other* **x** ∈ *S* *dominates* x* *. The set of all Pareto optimal solutions in the decision space is called Pareto-optimal set, or simply Pareto set (PS), and its image in the objective space is called Pareto-optimal front, or Pareto front (PF).*

**FIGURE 3:**
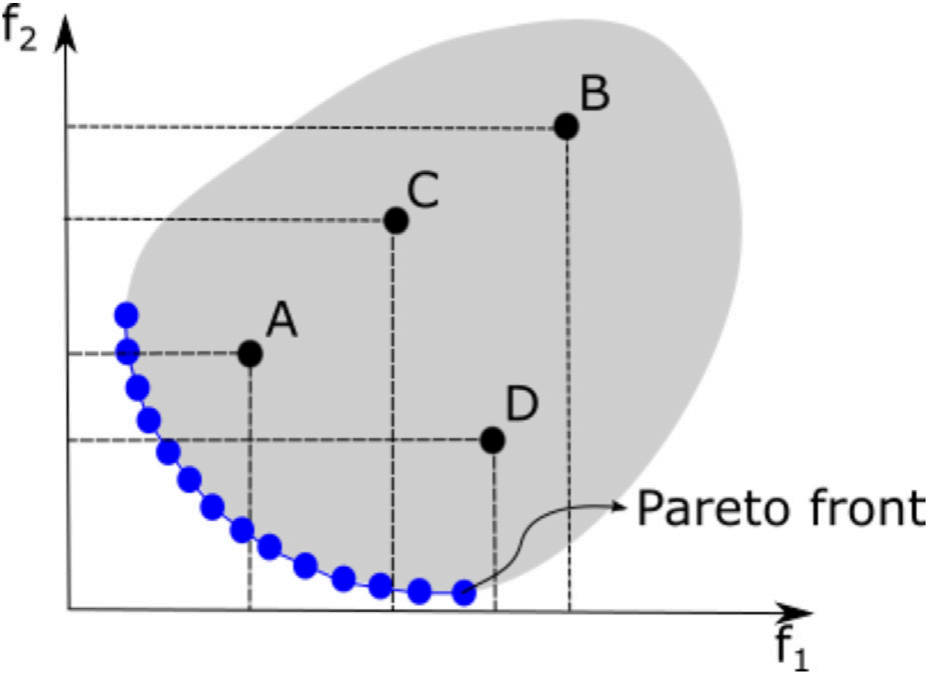
Pareto-optimal solutions for a two-objective minimization problem.

The Pareto-optimal set is the best collection of solutions to the MultiOOP. Research in the area of MultiOO is concerned with the problem of how to identify the PS or at least a good approximation of it. The ideal approach would be to find (i) a set of solutions as close as possible to the PF and (ii) as diverse as possible along that front Deb (2001). A typical PF is illustrated in Figure 3, where *f*_1_ and *f*_2_ are two objective functions that must be simultaneously minimized. Four different points (solutions values) are shown in this figure: solution *A* dominates solution *B*, as *f*_1_(*A*) < *f*_1_(*B*) and *f*_2_(*A*) < *f*_2_(*B*); *A* also dominates solution *C* for the same reason. However, solutions *A* and *D* are non-dominated by each other since *f*_1_(*A*) < *f*_1_(*D*) but *f*_2_(*A*) > *f*_2_(*D*). The Pareto-optimal solutions (dots in blue) that form the PF are those in which no objective can be improved without making at least one other objective worse.

The concept of dominance is of the utmost importance in this type of optimization problem, as it allows the comparison of two different solutions concerning distinct objective functions. Hence, to find the non-dominated set of solutions, the dominance relation ≺ is used to identify the best between two given solutions.

## 3 Solution methods

There are different ways to deal with a MultiOOP, usually consisting of three stages: model building, optimization, and decision-making Branke et al. (2008). First, we formulate the optimization problem in which the decision variables, objectives, and constraints are specified. Second, an optimization technique is used to find the best compromise solutions. Since the Pareto optimal solutions are equally acceptable, a decision maker (DM), who usually has expertise in the problem domain, must decide which solution(s) best suits your preferences. Following the approaches that distinguish when the DM interacts with the optimization procedure, the methods can be classified as Miettinen (1999):

• No-preference: no information about the importance of the objectives is assumed, and the DM’s preferences are not considered. The problem can be solved by any method used to find a single optimum solution. The solution obtained is presented to the DM, which will accept or reject it.
• *A priori*: the hopes and opinions of the DM are taken into consideration before the solution process. Usually, one preferred Pareto-optimal solution is obtained. Those methods, known as *preference-based*, require the DM to know the priority of each objective beforehand.
• *A posteriori*: the PS is first generated, and then the DM is supposed to select the most preferred solution from this set of alternatives. The preference information is considered after the optimization process.
• Interactive: the DM preferences are progressively used during the search procedure and are adjusted as the search continues.

The most intuitive and simple way to solve a MultiOOP is to convert it into a SingleOOP. The so-called *classical methods* mainly propose different ways of scalarising the objectives. These are commonly used methods that have existed for many decades. Most of them are *aggregation-based* techniques, which aggregate the objective functions into one objective. Only one Pareto-optimal solution can be found in these methods at each execution. To obtain different Pareto-optimum solutions, the SingleOOP must be solved several times, with different parameters for finding solutions in the entire Pareto-optimum region. The weighted sum, the *ɛ*-constraint, and the weighted Tchebycheff Miettinen (1999) methods are examples of such classical techniques. The weighted sum method is probably the most used classical approach due to its simplicity and ease of use. In this process, a weighting coefficient is associated with each objective function, and a weighted sum of the objectives is minimized. For each function *f*_*i*_, there is a weight *w*_*i*_ associated, such that 0 ≤ *w*_*i*_ ≤ 1 for all *i* = 1, …, *k*, with $\sum_{i=1}^{k}w_{i}=1$ (weights are normalised). By using this technique, the MultiOOP given in Eq. 1 is converted into a SingleOOP as follows

$$\begin{array}{cc} \text{Minimise} & \overset{k}{\underset{i=1}{\sum }}w_{i}f_{i}\left(\text{x}\right)\\ \text{subject to} & \text{x}\in S \end{array}$$ (2)

Considering that the ideal approach for solving MultiOOPs would be to find many different trade-off solutions as close as possible to the PF and as diverse as possible along that front, it becomes clear that classical methods need a great effort to meet these goals. As they combine multiple objectives into one, some knowledge of the problem is required. Note that the optimization of a single objective results in a single-point solution. In this way, multiple runs must be performed to generate different alternatives to the DM. Moreover, if the variable space is discontinuous and some objectives have many local minima, these methods may not work properly Srinivas and Deb (1994).

Differently, *multi-objective evolutionary methods* seek to optimize the problem in its original form, with independent objectives, providing in each execution a set of Pareto-optimal solutions. EAs have become extremely popular over the last years as a non-classical, stochastic search technique to solve multiOOPs. Next, we present a short overview of this class of methods and their main differences compared to the classical approaches.

### 3.1 Multi-objective evolutionary algorithms (MultiOEAs)

The process of evolution of species inspires EAs, and they differ from classical methods in various ways. The most prominent is using a population of candidate solutions instead of a single solution, as in classical methods. This characteristic allows those methods to find many Pareto-optimal solutions in a single run. Although they do not guarantee to find the optimal trade-off solutions, they can provide a satisfactory approximation set, which is (hopefully) not too far away from the true PF.

In the field of evolutionary computation, Genetic Algorithms (GAs) are the most popular ones. Developed by J. H. Holland in the 60s (Holland, 1962; Holland, 1975), they are based on the evolution of a population of individuals. Initially, a population of candidate solutions is randomly generated. For every individual, an objective function associates a fitness value indicating its suitability to the problem. At each iteration, individuals are selected to form the parents. Those parents are reproduced using different operators (*e.g.*, crossover, mutation) to generate new offsprings. Then, a replacement scheme is applied to determine which individuals of the population will survive from the offsprings and the parents. This process is repeated until a stopping criterion is reached. A general scheme of an EA is given in Algorithm 1.

Algorithm 1 Pseudocode of a general EA.

**1** Set *k* = 0;
**2** Randomly generate an initial population of solutions;
**3** Evaluate each solution in the initial population;
**4** **while** *k* < *G* **do**
**5** Selection of individuals;
**6** Apply mutation and crossover operators to obtain new solutions;
**7** Evaluate solutions;
**8** Select individuals for the next-generation;
**9** Set *k* = *k* + 1;

Following these baseline steps, GAs are known to be very efficient in solving real problems in several fields Slowik and Kwasnicka (2020). As they do not require additional information about the problem, like continuity or differentiability, they are also well-suitable to solve black-box optimization problems^1^.

The need to find trade-off solutions as close as possible to the Pareto optimal front (good convergence) and as diverse as possible along that front (good diversity) are the most important issues in MultiOOP. Therefore, search algorithms must be designed to obtain multiple solutions, each offering a different trade-off for the objective functions. In a simplistic case, this may be achieved by storing each found solution in an “archive” that maintains only non-dominated solutions. The way this archive is maintained, how individuals are selected and recombined, whether elitism is used or not, and how fitness assignment is applied characterize the various MultiOEAs. They can be classified into three main categories according to the different strategies employed Emmerich and Deutz (2018).

#### 3.1.1 Pareto-based

The earliest attempts for solving MultiOOPs with MultiOEAs were based on the Pareto dominance relation. The main idea is that the fitness value is assigned to individuals based on the Pareto-dominance principle to achieve good convergence. An explicit diversity preservation scheme is also employed to maintain the diversity of solutions. Some of the most representative techniques of this class of methods are the Strength Pareto EA (SPEA/SPEA-II) Zitzler and Thiele (1998, 1999); Zitzler et al. (2001), the Pareto Archived Evolution Strategy (PAES) Knowles and Corne (1999, 2000), the Niched Pareto GA (NPGA/NPGA2) Horn et al. (1994); Erickson et al. (2001) and the Non-dominated Sorting GA (NSGA/NSGA-II) Srinivas and Deb (1994); Deb et al. (2002).

The primary concern with the Pareto-based approach arises when the number of objectives increases, particularly beyond three objectives. In such cases, it becomes increasingly challenging for these techniques to select solutions, as most of the solutions in the population tend to be non-dominated by one another. Section 4 discusses some of the main difficulties that arise when the number of objectives increases.

#### 3.1.2 Indicator-based

In this approach, performance metrics, also known as quality indicators, are employed to define the selection mechanisms. Performance metrics are used to assess the quality of an approximation set generated by an algorithm. It assigns a real value to one or more approximation sets depending on certain quality aspects, such as (i) convergence toward the Pareto optimal region and (ii) diversity of solutions along the PF. The underlying idea of those techniques is to optimize the indicator value of the non-dominated set generated throughout the evolutionary process.

From the literature, it is not difficult to notice that the HV indicator is the most widely adopted metric for evaluating indicator-based optimizers and assessing non-dominated sets’ quality. This metric calculates the volume of the dominated region by the obtained solution. Although there are many efforts to reduce the computational complexity of the HV computation, it is also known that the high computational cost involved when the number of objectives increases could limit the use of this metric Beume et al. (2009); Guerreiro and Fonseca (2018). However, the theoretical properties of the HV justify its widespread acceptance Zitzler et al. (2003).

A recent survey on indicator-based MultiOEAs can be found in Falcón-Cardona and Coello (2020), where the authors presented solution methods from their origins up to their applications by current state-of-the-art approaches.

#### 3.1.3 Decomposition-based

The pioneering work on decomposition-based methods was due to Zhang and Li (2007), who first proposed in 2007 the Multiobjective EA Based on Decomposition (MOEA/D). In this approach, the MultiOOP with *k* objectives is decomposed into *M* single-objective subproblems through some aggregation technique. These *M* subproblems, represented by *M*-weighted vectors, are simultaneously optimized in a single run. Different weight vectors need to be used to generate a set of Pareto-optimal solutions. The MOEA/D explores the neighborhood relationship between these subproblems to solve the original problem in (1) efficiently.

A variety of methods was proposed for improving the design of the components of the MOEA/D, motivated by the existing limitations of the method. Later investigations tried to improve its performance by seeking new ways of, for instance, (i) decomposing the problem, (ii) generating the weight vectors, (iii) improving the efficiency of genetic operators, and iv) enabling the application of the method to ManyOOPs Trivedi et al. (2017); Xu et al. (2020).

## 4 Dealing with many-optimization problems

ManyOOPs are those having more than three objectives. As previously mentioned, drug design problems usually have more than three objectives to be optimized simultaneously, making them potential candidates for problems to be treated with ManyOO approaches.

### 4.1 Main challenges

**FIGURE 4:**
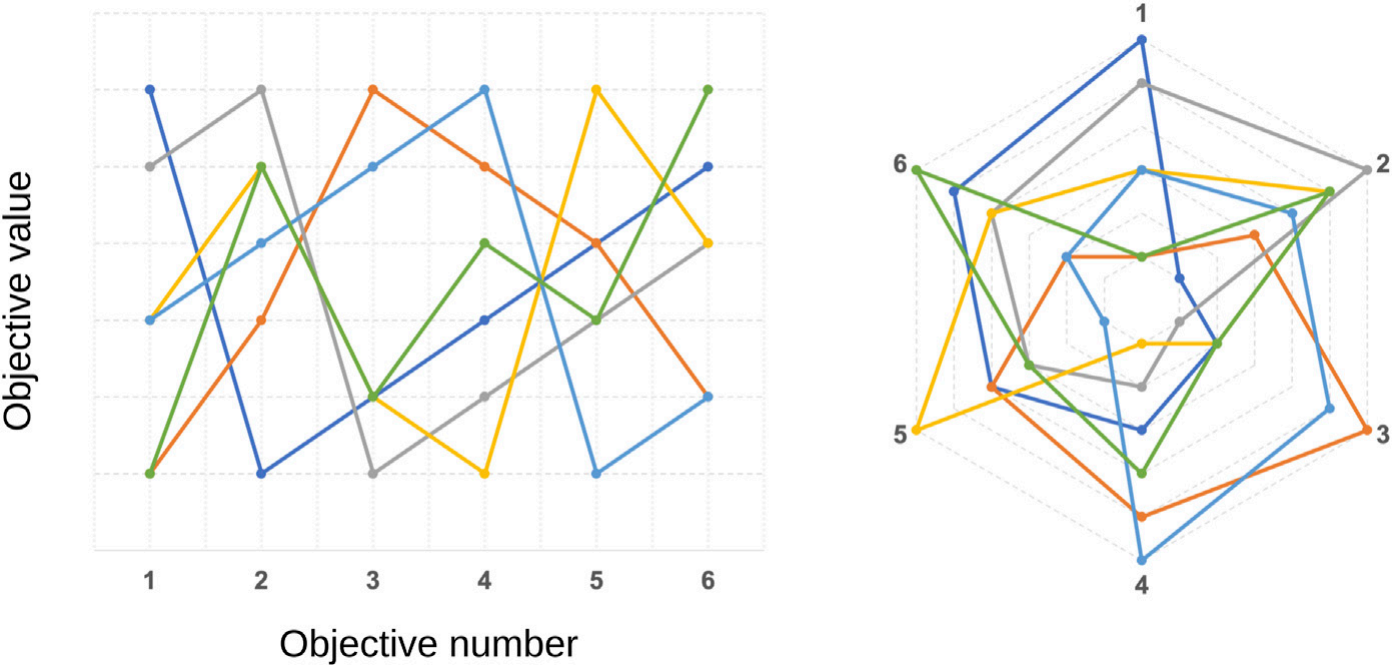
Illustration of parallel coordinates (left) and radar (right) plots for different solutions in a 6-objective optimization problem. Each coloured solid line represents one non-dominated solution.

The presence of many objectives introduces several challenges that affect the choice and the design of evolutionary techniques. The difficulty of optimizing a large number of objectives is due to the so-called *curse of dimensionality*. The PF takes different forms depending on the number of objectives, *e.g.*, a two-objective problem results in a PF that is a curve or line, while a three-objective problem generates a PF that is a surface. As the number of objectives increases beyond four, the PF may be represented by a hypersurface or other higher-dimensional shape. Therefore, the number of solutions needed to represent the entire PF grows exponentially with the number of objectives Saxena et al. (2013). When the number of objective functions increases, one has to deal with the following issues Ishibuchi et al. (2008):

• almost all solutions in the population become non-dominated by each other, leading to a phenomenon called *dominance resistance* Purshouse and Fleming (2007). Such characteristic severely deteriorates the convergence of MultiOEAs;
• scaling issues may be encountered in choosing the appropriate size of the population since the number of points needed to approximate the entire Pareto front increases exponentially with the number of objectives Saxena et al. (2013);
• beyond 3D space, visualizing the solution set in the objective space becomes less intuitive and needs special techniques, such as parallel coordinates or radar plots (Figure 4). Moreover, that makes it harder for the DM to choose the best solution.
• performance metrics are also affected by the curse of dimensionality, *e.g.*, the HV and the IGD metrics have their performance compromised Ishibuchi et al. (2016);
• classical aggregation techniques (presented in Section 3) are not limited to problems with up to three objectives. However, the difficulties pointed out in such methodology grow increasingly, *e.g.*, setting the weights of a large number of coefficients.

Given the difficulties mentioned above, the importance of treating ManyOOPs as a distinct class of problem is evident. In Allmendinger et al. (2022), the authors reflect on the question: “What if we increase the number of objectives?” In their paper, theoretical implications are presented on how the presence of many-objectives can impact the performance of MultiOEAs when solving the NK-landscapes problem Verel et al. (2013). They identified a series of drawbacks of dominance-based (e.g., NSGA-II Deb et al. (2002)), indicator-based (e.g., IBEA Zitzler and Künzli (2004)), and decomposition-based (e.g., MOEA/D Zhang and Li (2007)) MultiOEAs, along with recommendations for enhancing these techniques in the context of ManyOOPs. Thus, it is clear that MultiOEAs encounter performance limitations when applied to problems with more than three objectives. Therefore, evolutionary techniques that can be effectively applied to ManyOOPs are often referred to as ManyOEAs and represent the cutting edge of multi-objective optimization research. The following sections will cover different EAs strategies to deal with ManyOOPs efficiently.

### 4.2 Many-objective evolutionary algorithms (ManyOEAs)

Unsurprisingly, new methods have been proposed to improve existing MultiOO techniques, and new methodologies have been developed in recent years. For readers interested in exploring existing many-objective algorithms across various domains, we recommend referring to the extensive list provided in the articles Mane and Narasinga Rao (2017); von Lücken et al. (2019). We will reference a few relevant strategies and introduce more recently published approaches. Herein, we classified ManyOEAs into five categories according to the approach adopted to treat the problem in a ManyOO context: *relaxed dominance, indicator, decomposition, dimensionality reduction* and *hybrid techniques*. The Supplementary Material includes pseudocodes for some of the referenced methods.

#### 4.2.1 Relaxed dominance

As previously mentioned, when the number of objectives increases, almost all solutions in the population become non-dominated by each other. Hence, for MultiOEAs based on the Pareto dominance relation, the selection pressure towards the PF is compromised. A path explored to circumvent the scalability issue of dominance-based methods is to use relaxed forms of Pareto dominance to reduce the impact of dominance resistance. Such an approach can enhance the selection pressure toward the PF and provide a way of regulating the convergence of MultiOEAs.

**FIGURE 5:**
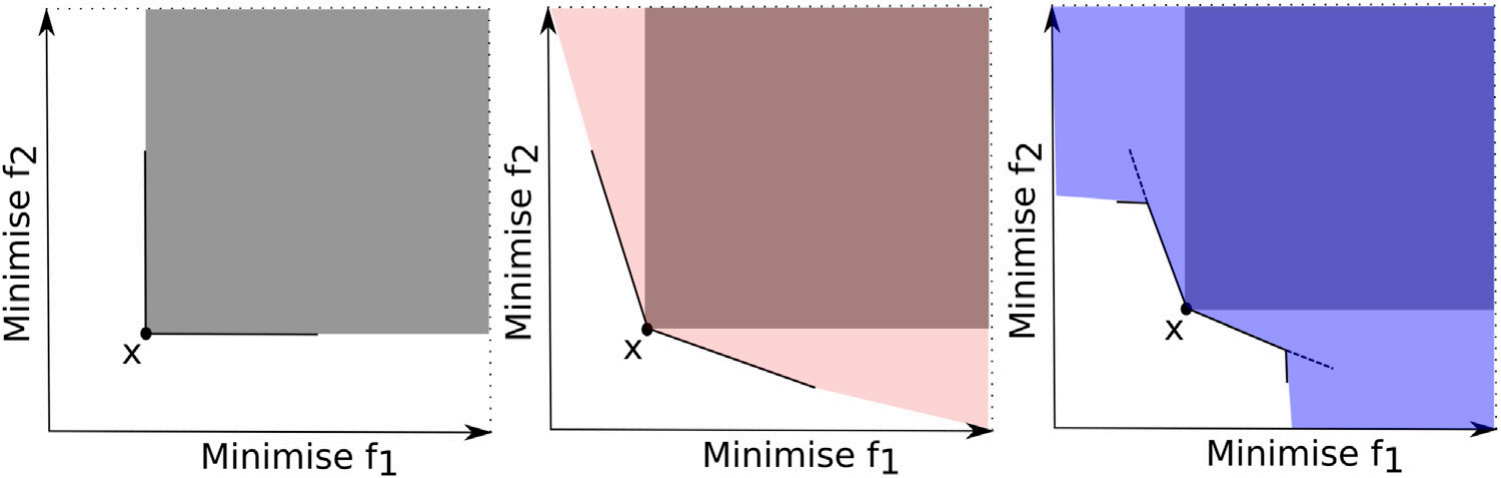
Illustration of dominated areas by solution *x* using three dominance relations: (left) Pareto dominance, (middle) *α*-dominance and (right) CN*α*-dominance.

Relaxed Pareto dominance (RPD) relations modify the Pareto dominance concept for better-discriminating solutions for selecting the best ones with enhanced selection pressure. The *α*-dominance Ikeda et al. (2001), *ϵ*-dominance Laumanns et al. (2002), cone *ϵ*-dominance Batista et al. (2011), *θ*-dominance Yuan et al. (2016), CN-dominance Dai et al. (2014), CN*α*-dominance Liu J. et al. (2019), and MultiRPD Zhu et al. (2022) are some examples of RPDs. Figure 5 illustrates the dominated area of a solution **x** using three different dominance relations: (a) the Pareto dominance, (b) the *α*-dominance and (c) the CN*α*-dominance for a bi-objective problem. Observe that the *α*-dominance and the CN*α*-dominance expand the domination area compared to the Pareto dominance. Under these relaxed definitions, a solution is expected to have a greater chance of being dominated by other solutions and the selection pressure towards the PF is enhanced.

In Li et al. (2015a), one can find diverse RPD-based methods, each applying a different RPD approach. Recently, the CN*α*-dominance proposed in Liu J. et al. (2019) combines two existing dominance relations, the *α*-dominance Ikeda et al. (2001) and the CN-dominance Dai et al. (2014). The idea was to strengthen the selection pressure by expanding the dominated area by combining two other RPDs, as shown in Figure 5. (c) Computational experiments considered a non-dominated neighbor Immune Algorithm (NNIA) as the baseline method. The NNIA with the proposed CN*α*-dominance was compared against the NNIA with four different dominance relations. The results demonstrated the superiority of the CN*α*-dominance against different dominance relations in terms of solution quality and selection pressure on benchmark problems with five to fifty-five objectives.

#### 4.2.2 Indicator

To improve the convergence ability of ManyOEAs, extensive research has been conducted on fitness evaluation mechanisms based on quality indicators (Falcón-Cardona and Coello, 2020). As mentioned previously, the high-dimensional problem encountered in ManyOO may turn the application of HV-based MultiOEAs impracticable despite the many efforts to mitigate its computational cost. The first method that attempted to accelerate the HV computation and make it scalable for a large number of objectives was proposed by Bader *et al.* Bader et al. (2010); Bader and Zitzler (2011). The main idea of the hypervolume estimation algorithm (HypE) was not to calculate the exact values of the HV but instead to provide an estimate of this value through Monte Carlo simulations. Experimental results showed that HypE achieved competitive performance regarding the average HV on benchmark problems with up to fifty objectives.

The Two-archive methodology (Two-Arch/Two-Arch2) Praditwong and Yao (2006); Wang et al. (2015) was the first to divide the non-dominated solution set into two archives, one that promotes convergence (CA) and another that emphasizes diversity (DA). CA and DA employ different updating rules that reflect their respective roles in the optimization process. In Two-Arch2, the update rule of CA is based on the quality indicator *I*_*ϵ*+_ from IBEA Zitzler and Künzli (2004), and the DA archive is updated based on the Pareto dominance rule. This method falls under the hybrid approach category. The results demonstrated that Two-Arch and Two-Arch2 outperformed other ManyOEAs in terms of convergence with comparable diversity quality in problems having two to eight objectives for the Two-Arch and up to twenty objectives for the Two-Arch2.

#### 4.2.3 Decomposition

In decomposition-based methods, the ManyOOP is divided into several SingleOO subproblems using a set of weight vectors. The basic idea is to find a set of well-distributed, non-dominated solutions along the PF using generated weight vectors so the diversity of the population is controlled explicitly by weight vectors. Ideally, each solution in the population is associated with a subproblem.

The most representative algorithms of this class are the MOEA/DD Li K. et al. (2015) and the NSGA-III Deb and Jain (2014); Jain and Deb (2014), which are extensions of the MOEA/D Zhang and Li (2007) and NSGA-II Deb et al. (2002), respectively. Those methods may also be classified as hybrid, employing a decomposition-based approach to maintain population diversity, while the Pareto dominance rule controls the algorithm’s convergence. Both methods outperform many contemporary MultiOEAs. It is worth noting that the performance of those methods strongly depends on the shape of the PF Ishibuchi et al. (2017).

The MOEA/D with Update when Required (MOEA/D-UR) method de Farias and Araújo (2022) proposed a new scheme to adapt the weight vectors depending on whether they show signs of convergence. An additional technique for partitioning the objective space was proposed to increase the spread of individuals in the population to estimate the level of regularity of the PF’s shape. The computational experiments show that the MOEA/D-UR has competitive performance compared to ten state-of-the-art ManyOEAs in test problems and real-world problems with up to fifteen objectives.

#### 4.2.4 Dimensionality reduction

**FIGURE 6:**
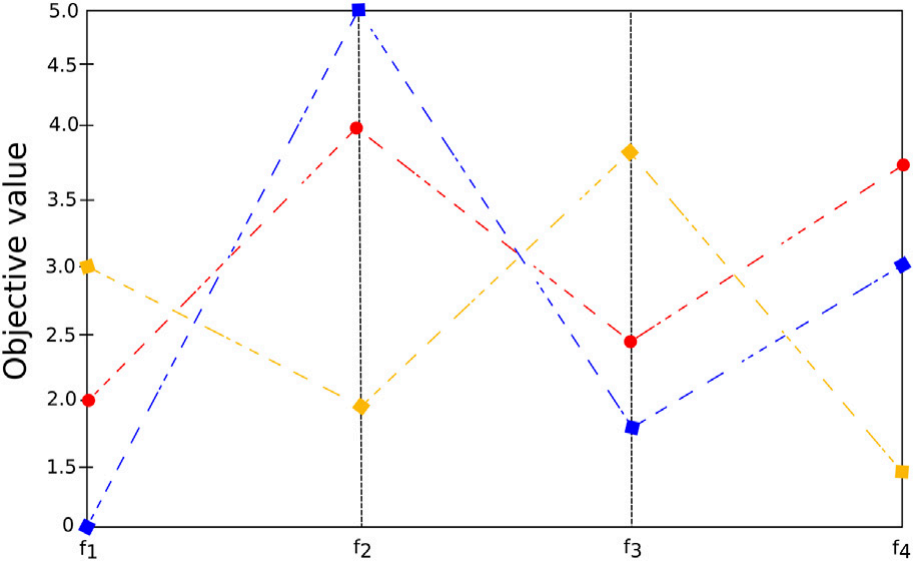
Parallel coordinate plot illustrating four objective values for three solutions with possible redundant objectives.

The visualization of objectives in large dimensional space can be difficult. See, for example, Figure 6 (a), in which seven objective values are presented for many different solutions (each represented by a color). The more pronounced the slope of the line connecting two solutions, the higher the likelihood of a possible conflict between them. In dimensionality reduction-based methods, the idea is to decrease the problem’s difficulty by reducing the number of objectives by identifying redundant objectives Brockhoff and Zitzler (2006, 2007). For many problems, a smaller set of *m* (*m* < *k*) conflicting objectives exist that can generate the same PF as the original problem Yuan et al. (2018). For instance, Figure 6 shows the parallel coordinates plot of three solutions *x*_1_ (blue), *x*_2_ (red) and *x*_3_ (yellow) and four objectives. The figure indicates that the objective functions *f*_1_ and *f*_3_ are redundant, since *f*_1_ (*x*_1_) < *f*_1_ (*x*_2_) < *f*_1_ (*x*_3_) as well as *f*_3_ (*x*_1_) < *f*_3_ (*x*_2_) < *f*_3_ (*x*_3_), that is, when *f*_1_ is minimized *f*_3_ is also minimized. Therefore, we can consider {*f*_1_, *f*_2_, *f*_4_} as the minimum objective set that preserves the dominance structure.

A list of many dimensionality reduction frameworks and ManyOEAs based on them is presented in Li B. et al. (2015), in which the methods are classified as *online* and *offline*. Online methods reduce the number of objectives gradually during the search process. In contrast, offline methods reduce the number of objectives after obtaining the PS. These methods can reduce the computational load of ManyOEAs and assist DMs in distinguishing points based on non-redundant objectives. However, an open question is whether the loss of information can cause problems in the optimization process.

A different approach for reducing the number of objectives was proposed by de Freitas et al. (2015), where the authors introduced the Aggregation Tree (AT). The AT tool allows the visualization of redundancies and conflict between objectives in the form of a tree by using the concept of *harmony* to reduce the number of objectives. The more harmonic the two objectives are, the more suitable they are to be aggregated into a group of objectives without much loss in the representation of the PS. This technique was used in a multi-objective GA, the GAPF algorithm, to solve a protein structure prediction problem in which the AT was used to arrange seven terms of the energy function into a three-objective problem Rocha et al. (2017).

#### 4.2.5 Hybrid approach

Hybrid techniques have been proposed to balance convergence and diversity in ManyOOPs. The method proposed in Zou et al. (2021), MaOEADRA, is based on dominance and decomposition approaches. An elitism mechanism is exploited to balance the convergence and diversity of the evolutionary process. Simultaneously, a reference point adaptation scheme is designed to “learn” the true PF shape of different problems. Computational results showed that the MaOEADRA outperformed seven state-of-the-art algorithms on various test problems with up to fifteen objectives.

The IDEA algorithm Xia et al. (2023) couples indicator-based and decomposition-based mechanisms. The decomposition-based approach promotes population diversity, while the $I_{\infty }^{r}$ Yuan et al. (2021) metric is used as the indicator value for achieving population convergence and distinguishing individuals. The performance of the method was evaluated on test problems with up to fifteen objectives. The results demonstrated that IDEA is effective in solving ManyOOPs.

## 5 Applications in *de novo* drug design

In the field of Computational Chemistry, MultiOO has been adopted for decades to obtain trade-offs among the objectives considered Handl et al. (2007); Ekins et al. (2010); Nicolotti et al. (2011); Parikh et al. (2023). A multitude of multi-objective approaches can be found in the literature, and we do not intend to describe or cite them exhaustively. This section describes some reported studies that explicitly apply multi-objective and many-objective strategies in dnDD. Following the previously defined classifications, the methodologies reviewed are mainly classified as aggregation-based and Pareto-based methods.

Recalling the preferences of the DM, the aggregation methods that will be cited in the next sections are mainly based on (i) *a priori* information, when the DM’s preferences are considered before the optimization process, and thus only a single final solution is generated, and (ii) *a posteriori* information, when a set of Pareto optimal solutions are generated for the DM to choose the best-suited solution.

### 5.1 MultiOO methods: optimizing up to three objectives

#### 5.1.1 Aggregation-based

The GANDI algorithm Dey and Caflisch (2008) is a fragment-based approach that generates molecules by joining pre-docked fragments with a list of fragments provided by the user. In the paper, GA Holland (1975) and Tabu Search (TS) Glover and Laguna (1997) algorithms are used in combination to match those fragments. While the GA was used to generate new fragments by applying genetic operators, the TS was adopted to link those encoded fragments. GANDI is an *a priori* method that scalarises the scoring functions into one, providing a single optimal solution. The weighted sum approach was used to aggregate three scoring functions to be minimized: a force field-based binding energy and two measures of similarity to a user-defined structure. Forbidden connections were avoided to prevent the generation of unstable molecules. When applied to the CDK2 kinase, the proposed method generated 1.809 molecules, of which eight were present in the ZINC database and commercially available Irwin and Shoichet (2005).

The method proposed by Devi et al. (2014) used a GA guided by the scalarisation of two objective functions, drug-likeness (by Lipinski’s Rule of 5 Lipinski (2004)) and similarity (by Tanimoto similarity Loving et al. (2010)) to a known reference molecule from the e-Drug3D database. The weights of the objectives are set *a priori*. Two experiments were conducted, one using a local anesthetic drug (lidocaine) and another using an anti-cancer molecule (furano-pyrimidine) as reference molecules. For each reference molecule, the authors analyzed the proposed method using one (Tanimoto similarity alone) and two objectives (using a weighted sum of the similarity and drug-likeness functions). For the furano-pyrimidine molecule, the two-objective version of the method could generate drug-like molecules more efficiently than the one-objective formulation.

The MoGADdrug method, introduced in Devi et al. (2021), is a fragment-based GA that constructs new molecules from a set of chemical fragments and a reference molecule as inputs. Building upon previous work Devi et al. (2014), which considered only two fragments (acid and amine), this method incorporates a variable-length representation to construct new solutions considering double amine fragments, thus allowing three fragment types. The objective function is a weighted sum of the oral bio-availability score Lipinski (2004), and the 2D similarity based on the Tanimoto coefficient Brown (2009), with the weights being set *a priori*. The MoGADdrug could design drug-like molecules similar to lidocaine, furano-pyrimidine derivative, imatinib, atorvastatin, and glipizide.

#### 5.1.2 Pareto-based

The Compound Generator (CoG) method Brown et al. (2004) represents the first instance of a MultiOEA being applied in dnDD. The authors used Genetic Programming (GP), in which the topology of molecules is represented by graphs, where the graph nodes represent the atoms. Special mutation and crossover operators were implemented to generate new molecules, and no constraints were imposed in the search space. The problem was formulated as a two-objective optimization problem, where they simultaneously maximize the evolved solutions regarding the Tanimoto similarity Bajusz et al. (2015) between the candidate molecule and two compounds representative of different chemical classes. The authors performed two experiments: one using two similar molecules and another using two diverse molecules. The results demonstrated that the graph-based CoG could generate a set of novel molecules that are visibly similar to the target compounds in both experiments.

Another graph-based framework, MEGA Nicolaou et al. (2009), tested the creation of new selective estrogen receptor (ER) compounds, aiming to maximize the docking score for the “positive” target receptor ER-*β* and minimize scores for the “negative” but closely related target ER-*α*. To generate molecules with desired chemical profiles similar to the known ligand tamoxifen, they used filters based on the Rule-of-Five (Ro5) and Tanimoto similarity scores as constraints. The authors pointed out the uneven nature of the objectives, *i.e.*, identifying solutions with reduced binding affinity for ER-*α* is substantially simpler than designing compounds with increased binding affinity for ER-*β*. This may lead to intense search space exploration towards the “easier” objective. To circumvent this situation, a mechanism called *niching* was used to preserve the diversity of the molecules, ensuring that the objectives were treated equally and there were no dominance conditions in favor of a specific objective. In addition, they applied *Pareto-elitism* to prevent good solutions from being “lost” during the generations. The authors reported that both mechanisms generated many non-dominated solutions while increasing the PF extension.

In Daeyaert and Deem (2017), synthesizable molecules were generated using a GA named Synopsis Vinkers et al. (2003). The authors proposed an improved version of the *de novo* program Synopsis that incorporates the non-dominated sorting procedure from NSGAII Deb et al. (2002). The proposed algorithm was evaluated to optimize two objectives: the docking scores, computed by the Autodock Vina program Trott and Olson (2010), associated with the fibroblast growth factor (FGFR) and the vascular endothelial growth factor (VEGFR), aiming to generate dual selective inhibitors for cancer. Moreover, thirteen additional scores were used as constraints to avoid generating unwanted molecules. The obtained solutions exhibit good predicted binding energies to their targets and possess structural and physicochemical parameters falling within the typical range for drug-like molecules. The use of the Pareto dominance approach enabled the generation of high-affinity compounds within the imposed restrictions, unlike the aggregated single-objective approach, which rarely produces good-quality solutions.

In the works of Devi et al. (2019, 2020), two multi-objective methods were explored for dnDD of new drug-like molecules: the monkey algorithm (MoMADrug) Devi et al. (2019) and the biofilm algorithm (MOBifi) Devi et al. (2020). MoMADrug is inspired by the behavior of monkeys, while MOBifi is inspired by the life cycle of bacteria in a biofilm. Both methods were adopted in the context of fragment-based *de novo* design and considered the Tanimoto similarity to known compounds Brown (2009) and oral-bioavailability scores Lipinski (2004) as the objectives to be maximized. In addition to these two objectives, MOBifi also employed the Veber score Veber et al. (2002) as a third objective related to oral bioavailability. In Devi et al. (2019), the authors compared the MoMADrug against the MoGADdrug Devi et al. (2021), which uses a weighted sum of the objectives. Their results showed that MoMADrug could produce a more diverse set of solutions due to its multi-objective nature and its use of Pareto dominance criteria.

The MOBifi Devi et al. (2020) was originally evaluated in three unconstrained benchmark problems from CEC 2009 Zhang et al. (2008) with three objectives against five other multi-objective methods. It was found that MOBifi is a competitive method in terms of the inverted generational distance (IGD) and the maximum spread (MS) performance metrics. The MOBifi method was applied to generate drug-like molecules based on reference anti-diabetic compounds from herbal plants. These generated molecules were further docked against the therapeutic targets tyrosine phosphatase 1B (PTP1B) and *α*-glucosidase (AGS) associated with the diabetes treatment. Two generated compounds exhibited docking scores similar to the reference inhibitor (rutin), indicating their potential as anti-diabetic agents. Since the fragments used in the dnDD strategy were generated from the commercially available databases Enamine and e-Lead3d, based on the predetermined coupling reactions concept Yuan et al. (2011), the designed molecules have a high probability of being successfully synthesized and purchased from the chemical vendors.

The proposed approach known as Deep Evolutionary Learning (DEL) introduced in Grantham et al. (2022) combines a deep generative model (DGM) with multi-objective evolutionary algorithms (MultiOEA) for dnDD. DEL leverages a fragment-based variational autoencoder (FragVAE) and NSGAII components, including non-domination rank and crowding distance, to design new molecules. By operating in the continuous latent representation space generated by the neural generative model, DEL avoids the limitations of a discrete structural space. The DGM is iteratively fine-tuned based on the newly generated populations of samples with better properties. The quantitative estimation of drug-likeness (QED), synthetic accessibility score (SAS), and logP were selected as objectives in DEL. Extensive validation of the approach was performed to assess its population validity, novelty, and diversity across various benchmarking sets, including those in the MOSES framework.

Based on the DEL framework, in Mukaidaisi et al. (2022), a graph-based DGM, called JTVAE (Junction Tree Variational AutoEncoder), is integrated into DEL to provide a latent representation space for the MultiOEA exploration. Unlike FragVAE, which utilizes SMILES fragmentation, the JTVAE employs graph fragmentation following the subgraph-by-subgraph strategy. Binding affinity score (BAS) predicted with the docking program QVina, SAS, and logP of the generated molecules are the three objectives to be optimized. During the optimization process, non-dominated ranking is performed. The VAE model is refined by selecting the high-quality generated molecules possessing significant BAS, SAS, and logP properties for the next-generation. Computational experiments were conducted comparing DEL + FragVAE with the JTVAE approach on the ZINC dataset and on a variant of ZINC that included drug molecules from the DrugBank database (ZINC + DrugBank dataset). The results demonstrated that both methods improved the properties of the molecules along the generations and that the JTVAE has a higher HV value than FragVAE. These experimental results confirm that the JTVAE approach, when compared to the FragVAE approach, improves the properties of molecules during the optimization process and leads to a higher HV value.

The DrugEx is a ligand-based approach that applies RNN-based reinforcement learning to generate new chemical structures in the SMILES format. The first version of DrugEx Liu X. et al. (2019) was designed to generate ligands by performing single-objective optimization using the predicted affinity against the human adenosine A_2*A*_ receptor as the objective through a random forest-based quantitative structure-activity relationship (QSAR) model.

In the second version, DrugEx v2 Liu et al. (2021) expanded its usage to include multi-objective optimization by applying the concept of mutation and crossover into the Reinforcement Learning (RL) framework and a Pareto ranking procedure to handle the different objectives. The performance of DrugEx v2 was compared to ORGANIC Sanchez-Lengeling et al. (2017) and REINVENT Olivecrona et al. (2017) methods by considering as objectives the affinity prediction against either multiple targets or a single target while considering off-target effects. For multiple targets, the desired molecules should exhibit a high affinity towards A_1_A and A_2*A*_A receptors. In contrast, in the target-specific scenario, the designed molecules should display high affinity towards A_2*A*_ while maintaining low affinity to A_1_. In addition, low affinity to the hERG channel (Ether-à-gogo-Related Gene, the *α* subunit of a potassium ion channel) is required in both cases. The generated molecules showed a large percentage of validity, low duplication, and similarity to known ligands.

In the latest version, DrugEx v3 Liu et al. (2023) adopted a graph-based transformer model as the generative model, considering user-defined scaffolds as inputs to create new molecules with desired chemical profiles. A novel encoding scheme for atoms and bonds was proposed based on an adjacency matrix to enable the transformer model to handle molecular graph representations. Unlike the previous versions, DrugEX v3 was evaluated considering two objectives: (i) the drug-likeness using the QED score and (ii) the affinity score towards the A2A receptor predicted with the random forest-based QSAR model. A Pareto-based ranking scheme was employed to rank molecules based on the average Tanimoto distance instead of the commonly employed crowding distance. SMILES and graph representations were tested on four deep learning (DL) architectures. According to the results, all the molecules generated by the newly proposed method using the provided scaffolds were valid, and most exhibited a high predicted affinity towards A_2*A*_.

### 5.2 ManyOO methods: dealing with more than three objectives

#### 5.2.1 Aggregation-based

In a recent study, Elend et al. (2022) enhanced a Pareto-based ManyOEA proposed in Cofala et al. (2020) by incorporating a neural language model trained on the ZINC database to improve the quality of generated molecules. The goal was to generate molecules that inhibit the therapeutic target Mpro of SARS-CoV-2 by considering multiple objectives such as predicted binding affinity (BA), quantitative estimate of drug-likeness (QED), natural product-likeness (NP), toxicity filter (TF), and synthetic accessibility (SA). A weighted sum approach was used to aggregate the objective values in which weights were set *a priori*. The proposed Evolutionary Molecular Generation Algorithm (EMGA) designed new molecules based on the SMILES representation. The transformer architecture in the neural language model was used as a mutation operator to generate molecule fragments, while a (*μ* + *λ*) Evolutionary Strategy (ES) was employed to perform a randomized search in the molecular structure search space. From the molecules generated by EMGA, twenty-one chemically valid molecules were selected for molecular dynamic (MD) simulations. Among them, two were identified as stable and had the potential to inhibit Mpro.

The ATOM Generative Molecular Design (ATOM GMD) proposed in McLoughlin et al. (2023) involves a two-stage process of a variational autoencoder (VAE) and a many-objective GA operating in the latent space. The VAE is used to map molecular structures to the continuous latent space. Specifically, a JTVAE maps a population of structures to a learned continuous latent space. The encoder component of the JTVAE converts each SMILES structure into a continuous latent vector, and the decoder component performs the opposite transformation. The GA-based approach searches for optimal molecules in the latent space, employing crossover and mutation operations to generate new molecules with desirable properties. The cost function of this ManyOO approach is a weighted sum of terms based on 12 predicted properties, including efficacy and safety (binding affinities against the therapeutic target histamine H1 receptor and the off-targets M2 and hERG predicted with QSAR models), pharmacokinetic profile (Ro5), and developability properties (SAS). The weights are set *a priori*. Focusing on developing potent and selective H1 antagonists, the ATOM GMD approach generated several molecules, of which 106 compounds were further synthesized and experimentally evaluated. Six tested compounds were found to bind H1 at nanomolar concentrations selectively. During the training, the authors included molecules from Neurocrine Biosciences, ChEMBL, and GoStar with known affinity against H1 and compounds structurally similar to the known H1 antagonists from the Enamine REAL Space. This was done to bias the generation of new structures towards the desired profile. Despite being a preprint at the time of our review, to the best of our knowledge, this is the first published work that successfully applied ManyOO, EAs, and ML methods with experimental validation. This highlights the powerful application of Multi/ManyOO strategies in the context of dnDD.

#### 5.2.2 Pareto-based

Recently, Verhellen (2022) evaluated the performance of graph-based implementations of NSGA-II and NSGA-III against a weighted sum method on case studies from the GuacaMol benchmark suite for dnDD Brown et al. (2019) and from datasets constructed to simulate polypharmacology scenarios. The number of optimized objectives ranged from four to five, depending on the benchmark molecule analyzed, including molecular weight, logP, similarity, affinity to a target, and/or blood–brain–barrier permeability. The optimization procedure removed undesired molecules from the population based on structural ADMET filters. Both approaches outperform the weighted sum method in terms of the HV indicator. Regarding efficiency, NSGA-III outperformed NSGA-II by performing fewer function evaluations in all benchmarks. However, both approaches showed similar performance on the analyzed chemical benchmarks. By conducting this comparison, the authors provided valuable insights for effectively applying Multi/ManyOO approaches in dnDD.

Molecules for anti-breast cancer were produced in Mei and Wu (2022) using an enhanced ManyOEA variant, AGM-MOEA Panichella (2019). The proposed method applies the crossover operator from Differential Evolution Price et al. (2005), non-dominated sorting from NSGA-II Deb et al. (2002), and a normalization method from NSGA-III Deb and Jain (2014) for generating and selecting molecules. Six objectives were simultaneously optimized: pIC_50_ and ADMET properties (Caco-2, CYP3 A4, hERG, HOB, MN), and three performance metrics measured the quality of the PF obtained. To highlight the importance of adopting a ManyOO approach, the authors demonstrated the conflicting nature of the objectives and emphasized that a simple aggregation of the objectives could be a difficult task. A comparative analysis was conducted between the proposed framework and three other methods: NSGA-II, NSGA-III, and the original AGE-MOEA. The results indicated the superiority of the proposed method regarding the performance metrics.

Cofala et al. (2020) proposed a method that combined a (*μ* + *λ*) ES and an NSGA-II-based method to design an inhibitor for SARS-CoV-2’s main protease. The ManyOEA proposed uses the SELFIES representation for designing new molecules. Five molecular properties were used in the optimization process: binding affinity (BA) computed by the QuickVina2 binding scoring function, QED, SAS, natural product-likeness (NP), and medical chemical filters (MC). Two experiments used the N3 inhibitor and lopinavir, an HIV main protease inhibitor, as ligands targeting the main protease Mpro receptor. First, a SingleOO experiment was conducted, where the fitness function was composed of a weighted sum of the five properties. In preliminary experiments, the authors observed that (i) molecules with high binding scores suffer from low QED scores, and (ii) defining the weights for the objectives could be difficult, concluding that a many-objective approach might be more appropriate. Hence, a many-objective analysis was also conducted, in which the HV metric was used to evaluate the final set of solutions. For the SingleOO experiment, the authors highlighted the conflicting nature of the objectives, which resulted in a trade-off between QED and NP scores *versus* SA and BA scores. Even so, according to the binding scores, the best molecule found in the SingleOO approach achieved better scores than those of N3 and lopinavir. On the other hand, the ManyOO provided a higher diversity of molecules when compared with the SingleOO case, achieving satisfactory values for all properties.

A Generative Adversarial Network (GAN), proposed by Abbasi et al. (2022), combines an autoencoder with a GAN to convert SMILES strings into latent space vectors and use them as real data in GAN training. To generate molecules that exhibit multiple desired properties, an optimization step based on feedbackGAN Gupta and Zou (2019) is applied, incorporating the NSGA-II method to generate non-dominated solutions that will be included in the training set. The case study analyzed aimed to find ligands that bind both to the Kappa Opioid Receptor (KOR) and the A2*A* receptor (ADORA2A). The other properties to be optimized include the binding affinity pIC50, the molecular topological polar surface area (TPSA), the solubility (LogP), and the SAS. The strategy adopted was evaluated based on several metrics, such as validity, uniqueness and novelty. The proposed framework generated molecules with a high level of diversity (over 0.88) and 100% uniqueness but a low percentage of validly generated molecules (30.2%).

### 5.3 Objectives or constraints?

In implementing different dnDD strategies, determining which of the desired properties should be considered as objectives or constraints is a very important aspect. Nicolaou et al. (2009) classified their objectives as primary (objectives used to guide the search procedure) and secondary (objectives acting as constraints that can be used as filters to restrict the search space). Properties serving as objectives are typically related to the similarity to a known ligand (*e.g.*, Tanimoto similarity) or binding affinity scores with one or more receptor target(s) of interest. Constraints usually involve descriptors related to pharmacokinetic prediction (*e.g.*, Lipinski’s Ro5) and synthetic accessibility. However, it is important to highlight that the researchers define the premises of development, not existing a rule to state which properties must be defined as an objective or a constraint.

Binding affinity scores are widely used as an objective in the optimization procedure to consider the potency of a compound against the therapeutic targets of interest. However, ignoring other information (objectives) important for a lead compound to pass clinical trials and reach the market can affect the quality of the designed molecules Fu et al. (2022). Therefore, several works also include pharmacokinetics-related descriptors in a Multi/ManyOO context, such as QED, logP and SAS.

As previously mentioned, when the number of objectives increases ( > 3), the number of Pareto-optimal solutions becomes intractable. Hence, in selecting the number of objectives, the researchers must also consider this issue when choosing an appropriate technique to deal with the different objectives and constraints. To alleviate this drawback, in Daeyaert and Deem (2017), it was proposed to define some objectives as constraints so that the dimension of the problem could be restricted to two or three objectives, allowing the application of MultiOEAs as the search procedure. On the other hand, many works adopted a scalarization procedure to aggregate the many objectives into one, using an *a prior* optimization method, thus transforming a multi-objective problem into a single-objective optimization process. In both approaches, detailed knowledge of the problem is required to define the most suitable properties that should be used as objectives or to set the weight vectors to obtain a Pareto-optimal solution (hopefully) in a desired region of the objective space.

It is also crucial to avoid the use of redundant objectives that would increase the complexity of the problem without providing a real increase in accuracy. For example, in the MOBifi Devi et al. (2020), they adopted two correlated objectives for drug-likeness (Veber and Lipinski’s rules). Despite these metrics adopts different parameters, the Veber’s rule is proposed as an improved model for oral bioavailability, thus probably not being conflicting with the Lipinski’s rule. As already described elsewhere, the MultiOO approaches aim to optimize only conflicting objectives. Therefore, the selection of the objectives can also be guided by dimensionality reduction methods to reduce the number of effective objectives, discarding correlated metrics.

EAs are originally unconstrained search techniques, requiring additional mechanisms to deal with constraints. A simple approach is to discard unwanted solutions during the search process. However, this strategy may not be adequate since the usual assumption is that the first generations of EAs may contain diverse infeasible but still promising solutions. Some examples of alternative approaches to deal with constraints include Mezura-Montes and Coello (2011); Rahimi et al. (2023):

• penalty functions to add a penalty value to the objective function for infeasible solutions;
• move operators and special representation schemes that guarantee the generation of feasible solutions;
• repair techniques to move unfeasible points back to the feasible space;
• feasibility rules for selecting feasible and infeasible individuals; and
• hybrid approaches that combine different strategies to treat constraints

Among those techniques, special move operators and repair techniques are used mainly to guarantee the generation of feasible solutions. For instance, the GANDI Dey and Caflisch (2008) methodology enforces feasibility by forbidding certain connections on the molecule. CoG Brown et al. (2004) applies special mutation and crossover operations on the graph representation. Synopsis Daeyaert and Deem (2017) enforces feasibility rules in which constraint violations are considered for selecting a new compound. Invalid and toxic molecules are discarded in DEL Grantham et al. (2022) and during the optimization process of EMOA Cofala et al. (2020). A repair mechanism is used in DrugEx v3 Liu et al. (2023) to correct chemically invalid molecules.

Recently, a helpful tool proposed by Schoenmaker et al. (2023) for molecular correction through a generative deep learning method could be used as a repair technique to correct invalid but interesting compounds.

Table 1 summarises the multi-objective and many-objective techniques cited in the previous sections, including their choice of objectives and constraints, the constraint handling techniques applied, and the Multi/ManyOO approach adopted.

**TABLE 1:** Multi-objective and many-objective methods for *de novo* drug design.

Method	Molecular generation	Objectives	Constraints	Constraints handling technique	Multi/ManyOO approach (final result)	REF.
Multi-objective methods						
GANDI	GA *^b^* and TS *^b^*	Force field-based binding energy and 2D/3D similarity measure to a known ligand	Chemical validity	Special move operators and elimination of compounds with steric clashes	Aggregation-based (single solution)	Dey and Caflisch (2008)
MOGA	GA *^b^*	Oral bio-availability and Tanimoto similarity with known ligands	Synthetic accessibility	Special move operators	Aggregation-based (single solution)	Devi et al. (2014)
MoGADdrug	GA *^b^*	Oral bio-availability and Tanimoto similarity with known ligands	Synthetic accessibility	Special move operators	Aggregation-based (single solution)	Devi et al. (2021)
CoG	GP *^b^*	Tanimoto similarity between two compounds	Chemical validity	Special move operators	Pareto-based	Brown et al. (2004)
MEGA	GA-based *^b^*	Docking score against multiple targets	Tanimoto similarity with known ligands and Ro5	Special move operators	Pareto-based	Nicolaou et al. (2009)
Synopsis	GA *^b^*	Docking score against multiple targets	RotB, longest sp3-sp3 chain, reactive groups, volume, logP, PSA, molar refractivity, interaction with specific regions of the targets (hinge, backpocket, hydrophobic)	Feasibility rules	Pareto-based	Daeyaert and Deem (2017)
MoMADrug	Monkey Algorithm	Tanimoto similarity with known ligands and Ro5	-	-	Pareto-based	Devi et al. (2019)
MOBifi	Biofilm Algorithm	Tanimoto similarity with known ligands, Ro5 and Veber rules	-	-	Pareto-based	Devi et al. (2020)
DEL	DGM *^b^* (VAE)	QED *^a^*, SAS *^a^* and logP	Chemical validity	Elimination of invalid and duplicated SMILES strings	Pareto-based	Grantham et al. (2022)
DEL-based	DGM *^b^* (JTVAE)	Affinity prediction, SAS *^a^* and logP	Chemical validity	Elimination of invalid and duplicated SMILES strings	Pareto-based	Mukaidaisi et al. (2022)
DrugEx v2	DRL *^b^*	Affinity prediction towards two targets and one off-target	-	-	Pareto-based	Liu et al. (2021)
DrugEx v3	Graph Tansformer	QED *^a^* and affinity prediction to a pre-defined receptor	Chemical validity	Repair technique	Pareto-based	Liu et al. (2023)
Many-objective methods						
EMGA	NLM *^b^* (Transformer)	Predicted affinity, QED *^a^*, natural product-likeness, toxicity scores and synthetic accessibility	-	-	Aggregation-based (single solution)	Elend et al. (2022)
ATOM-GMD	DGM *^b^* (JTVAE)	12 predicted properties, including terms related to efficacy and safety (on and off-target affinities predicted with QSAR models), Ro5 and accessibility scores	-	-	Aggregation-based (non-dominated solutions)	McLoughlin et al. (2023)
NSGAII and NSGAIII-based	GA-based *^b^*	Ranged from four to five (molecular weight, logP, similarity, affinity prediction to a target of interest, and/or blood–brain-barrier permeability)	ADMET filters	Elimination of undesirable compounds	Pareto-based	Verhellen (2022)
AGM-MOEA	EA-based *^b^*	Affinity prediction and ADMET properties (Caco-2, CYP3 A4, hERG, HOB, MN)	-	-	Pareto-based	Mei and Wu (2022)
EMOA	EA-based *^b^*	Affinity prediction, QED *^a^*, natural product-likeness, toxicity scores and SAS *^a^*	Toxicity filters	Elimination of toxic molecules	Pareto-based	Cofala et al. (2020)
GAN-based	GAN *^b^*	Affinity prediction, topological polar surface area (TPSA), LogP and SAS *^a^*	-	-	Pareto-based	Abbasi et al. (2022)

^a^ QED, Quantitative estimation of drug-likeness; SAS, Synthetic accessibility score.

^b^ GA, Genetic Algorithm; TS, Tabu Search; GP, Genetic Programming; EA, Evolutionary Algorithm; DGM, Deep Generative Model; GAN, Generative Adversarial Network; DRL, Deep Reinforcement Learning; NLM, Neural Language Model.

It is important to highlight that even considering only objectives, such as machine learning predicted binding affinities, related to diverse targets through many-objectives methodologies in dnDD could *per se* provide a robust framework for designing and optimizing more effective drug candidates. In fact, there is a crescent interest in the development of new compounds targeting multiple targets in a polypharmacology context (*on*-targets), for example, to reduce bacterial resistance, whereas avoiding the interaction with receptors related to side effects (*off*-targets) Zieba et al. (2022); Raghavendra et al. (2018); Wang H. et al. (2021); Dias et al. (2017). Furthermore, by incorporating diverse objectives and constraints related to potency, selectivity, and pharmacokinetics, ManyOO methodologies might enable the design of compounds that exhibit improved overall performance and a higher probability of success in clinical trials.

## 6 Discussion

The application of multi-objective techniques has become well-established in the field of dnDD. This strategy improves the solutions’ quality by considering multiple objectives simultaneously, leading to a more realistic representation of the problem. It is worth noting that a few papers Luukkonen et al. (2023); Liu et al. (2021) emphasize the importance of addressing problems with more than three objectives in diverse areas. However, the proper exploration of ManyOO approaches in dnDD is still scarce despite its significant potential.

The issue of dimensionality in dnDD has been recognized by researchers as an important topic that needs consideration Nicolaou et al. (2009); Devi et al. (2014); Grantham et al. (2022). Despite the use of ManyOEAs in Mei and Wu (2022); Verhellen (2022), none of these papers explicitly address the significantly more complex and challenging nature of problems that arise when dealing with more than three objectives. It is relevant to observe that in Cofala et al. (2020), five objectives are employed in the optimization process using the NSGA-II method, a MultiOEA more suitable for handling problems with up to three objectives. As previously mentioned, NSGA-II encounters additional difficulties as the number of objectives increases. In such scenarios, NSGA-III or other ManyOEAs could be more appropriate alternatives for handling the problem. Hence, it is important to highlight the following reflections.

1. ManyOO methods still have not received enough attention from the dnDD community. Among all the papers cited herein, only a few of them Elend et al. (2022); McLoughlin et al. (2023); Verhellen (2022); Mei and Wu (2022); Cofala et al. (2020) simultaneously optimized more than three objectives. One is a preprint, and the others were recently published. Although dnDD research has increased rapidly over the years, its application to the many-objective case seems recent.
2. Although ManyOEAs were used in those papers, rarely is there an emphasis on the fact that they are dealing with a much more complex and challenging class of problems compared to cases with up to three objectives.
3. We observed that many papers adopt aggregation-based techniques, most of which apply the weighted sum aggregation approach. It is important to note that other aggregation-based techniques are more efficient and equally simple to implement. The assumption that the optimization problems are always convex to justify the efficient use of weighted sum aggregation is not always valid in a complex and challenging problem such as the dnDD.
4. Some papers considered multi-target evaluations in the context of polypharmacology and/or off-targets, but they are often limited to two or three targets simultaneously. However, there are important contexts in which binding affinity against multiple targets could be considered at the same time, for example, when developing new compounds against infectious diseases (multiple targets for the same pathogen could improve drug efficacy) or in the case of kinase inhibitors (off-targets panel containing dozens of kinases to evaluate selectivity).
5. The main purpose of Multi- and ManyOO methods is to generate trade-off solutions considering conflicting objectives, not to improve the predictions of the objectives independently. For that reason, as in the single-objective problems, one should care about the accuracy of the adopted methods for calculating/predicting the objective values.

Aggregation-based and weight-based approaches are widely used because they simplify the problem by reducing its dimensionality. However, it is important to highlight that methods based on *a priori* aggregation provide only one solution among the several possibilities of existing non-dominated solutions. As stated earlier, using aggregating techniques is the easiest way to approach the problem, but it oversimplifies and fails to inform the user about the trade-offs between the objectives. Besides, with the increase in the number and diversity of objectives, choosing and normalizing weight vectors becomes progressively difficult. Thus, to overcome these issues and take advantage of a more diverse set of non-dominated solutions, we believe many-objective Pareto-based approaches will increase prevalence in dnDD and be the center of novel methodological developments. We also are convinced that, given its intrinsic attributes—namely, the quantity and variety of objectives and constraints, along with the diversity of possible approaches in their prioritization—many-objective dnDD optimization might indeed serve as a source of inspiration for new developments in more fundamental and general methodological frameworks within the field of ManyOO.

Although this paper does not focus on machine learning (ML) techniques, it is important to mention that recent papers have demonstrated a notable increase in the number of works incorporating ML techniques and evolutionary computing algorithms and concepts in their models, particularly in generative models. For those interested in this field, comprehensive reviews of ML techniques for dnDD can be found in Mouchlis et al. (2021); Bilodeau et al. (2022); Wang et al. (2022); Luukkonen et al. (2023). While few of them briefly mention papers exploring MultiOO, with the majority utilizing MultiOEAs, only the recently published paper Luukkonen et al. (2023) gives special attention to ML methods for multi-objective dnDD. They also list multi-objective ML-based methods, including EAs, reinforcement/conditional learning, and recurrent neural networks. We believe new developments involving ML techniques and ManyOEAs should generate powerful tools for dealing with dnDD.

## 7 Conclusion

This work presented an overview of MultiOO and ManyOO approaches applied in dnDD, particularly those based on evolutionary computation and machine learning techniques. We provide a general review of the definitions involved in MultiOOPs and ManyOOPs, emphasizing the main challenges that appear when the number of objectives of an optimization problem increases. Our review could trace possible improvements and drawbacks in designing new optimization techniques by examining how the molecular properties are utilized in the dnDD problem to define objectives and constraints.

The increasing interest in applying ManyOO in dnDD is evident, in which evolutionary computation, coupled with ML methods, has continuously strengthened. Those approaches evolve toward efficiently solving the dnDD problem regarding the number of objectives and/or constraints considered. Still, given the multitude and diverse dnDD’s characteristics as a ManyOO process, it may serve as a catalyst for new developments in more fundamental and general methodological frameworks within the ManyOO field.

Finally, integrating multi-target drug development and many-objectives optimization approaches has great potential for accelerating the discovery of innovative and more efficacious drug therapies.

## Footnotes

1 In black-box optimization, the objective function and/or constraints are approximated by computational methods that provide an answer (output) to the problem, given a set of input values. In practice, they often occur when the computation of the objective functions and/or constraints requires an expensive computer simulation or when their functional form are unknown.
