# Supplementary material for: Multi-and many-objective optimization: present and future in de novo drug design
Source: Front Chem. 2023 Dec 18;11:1288626. doi: 10.3389/fchem.2023.1288626 (PMC10773868; doi:10.3389/fchem.2023.1288626)
Supplement: Supplementary file 1 [file DataSheet1.PDF]

# Multi- and Many-Objective Optimization: Present and Future in *de novo* Drug Design
# – Supplementary Material –

Jaqueline S. Angelo, Isabella A. Guedes,
Helio J. C. Barbosa and Laurent E. Dardenne

## 1 Pseudocodes of many-objective EAs

This section provides brief pseudocodes for seven many-objective EAs cited in the paper, which are: NNIA [Liu et al., 2019], Two-Arch [Praditwong and Yao, 2006], NSGA-III [Deb and Jain, 2014], MOEA/DD [Li et al., 2015], GAPF [Rocha et al., 2017], MaOEADRA [Zou et al., 2021], and IDEA [Xia et al., 2023].

**Algorithm 1:** Pseudocode of the NNIA Algorithm [Liu et al., 2019].

**Input**: $Gmax$ (maximum number of generations);
$nD$ (maximum size of Dominant Population);
$nA$ (maximum size of Active Population);
$nC$ (size of Clone Population);
**Output**: $D_{Gmax+1}$ (final approximate Pareto-optimal set);

*Step1:* **Initialization:** Generate an initial antibody population $B_0$ with the size $n_D$. Create the initial $D_0 = \emptyset$, $A_0 = \emptyset$, and $C_0 = \emptyset$. Set $t = 0$.;

*Step2:* **Update Dominant Population:** Identify dominant antibodies in $B_t$; Copy all the dominant antibodies to form the temporary dominant population (denoted by $DT_{t+1}$); If the size of $DT_{t+1}$ is not greater than $n_D$, let $D_{t+1} = DT_{t+1}$. Otherwise, calculate the crowding-distance values of all individuals in $DT_{t+1}$, sort them in descending order of crowding-distance, choose the first $n_D$ individuals to form $D_{t+1}$.;

*Step3:* **Termination:** If $t \geq Gmax$ is satisfied, export $D_{t+1}$ as the output of the algorithm, Stop; Otherwise, $t = t + 1$.;

*Step4:* **Nondominated Neighbor-based Selection:** If the size of $D_t$ is not greater than $n_A$, let $A_t = D_t$. Otherwise, calculate the crowding-distance values of all individuals in $D_t$, sort them in descending order of crowding-distance, choose the first $n_A$ individuals to form $A_t$.;

*Step5:* **Proportional Cloning:** Get the clone population $C_t$ by applying the proportional cloning to $A_t$.;

*Step6:* **Recombination and Hypermutation:** Perform recombination and hypermutation on $C_t$ and set $C'_t$ to the resulting population.;

*Step7:* Get the antibody population $B_t$ by combining the $C'_t$ and $D_t$; go to Step2.;

**Algorithm 2:** Pseudocode of the Two-Arch Algorithm [Praditwong and Yao, 2006].

Initialize the population;<br>
Initialize archives to the empty set;<br>
Evaluate initial population;<br>
Set $t = 0$;<br>
**repeat**<br>
    Collect non-dominated individuals to archives;<br>
    Select parents from archives;<br>
    Apply genetic operators to generate a new population;<br>
    Evaluate the new population;<br>
    $t = t + 1$;<br>
**until** $t == MAX\_GENERATION$;

**Algorithm 3:** Pseudocode of NSGA-III Algorithm [Deb and Jain, 2014].

**Input:** $H$ structured reference points $Z^s$ or supplied aspiration points $Z^a$, parent population $P_t$;<br>
**Output:** $P_{t+1}$;<br>
$S_t = \emptyset, i = 1$;<br>
$Q_t$ = Recombination+Mutation($P_t$);<br>
$R_t = P_t \cup Q_t$;<br>
$(F1, F2, ...)$ = Non-dominated-sort($R_t$);<br>
**repeat**<br>
    $S_t = S_t \cup F_i$ and $i = i + 1$;<br>
**until** $|S_t| \geq N$;<br>
Last front to be included: $F_l = F_i$;<br>
**if** $|S_t| = N$ **then**<br>
    $P_{t+1} = S_t$, break<br>
**else**<br>
    $P_{t+1} = \cup_{j=1}^{l-1} F_j$;<br>
    Points to be chosen from $F_l : K = N - |P_{t+1}|$;<br>
    Normalize objectives and create reference set $Z^r$: $Normalize(f^n, S_t, Z^r, Z^s, Z^a)$;<br>
    Associate each member $s$ of $S_t$ with a reference point: $[\pi(s), d(s)] = Associate(S_t, Z^r)$ % $\pi(s)$: closest reference point, $d$: distance between $s$ and $\pi(s)$;<br>
    Compute niche count of reference point $j \in Z^r : \rho_j = \sum_{S \in S_t/F_l}((\pi(s) = j) \ ? \ 1 : 0)$;<br>
    Choose $K$ members one at a time from $F_l$ to construct $P_{t+1} : Niching(K, \rho_j, \pi, d, Z^r, F_l, P_{t+1)}$;

**Algorithm 4:** Pseudocode of MOEA/DD Algorithm [Li et al., 2015].

**Output:** population $P$;<br>
$[P, W, E] \leftarrow INITIALIZATION()$ ; `/*` $P$ `is the parent population,` $W$ `is the weight vector set and` $E$ `is the neighborhood index set */`<br>
**while** *termination criterion is not fulfilled* **do**<br>
    **for** $i \leftarrow 1$ **to** $N$ **do**<br>
        $P \leftarrow MATING\_SELECTION(E(i), P)$;<br>
        $S \leftarrow VARIATION(\overline{P})$;<br>
        **foreach** $x^c \in S$ **do**<br>
            $P \leftarrow UPDATE\_POPULATION(P, x^c)$ ; `/*` $x^c$ `is an offspring */`<br>
**Return:** $P$

**Algorithm 5:** Pseudocode of GAPF Algorithm [Rocha et al., 2017].

Generate initial population;
Evaluate the population (using the GAPF energy potentials);
**for** $t = 0$ **to** $NEvalsMax$ **do**
    Parental selection tournament;
    Apply genetic operator on parents;
    Evaluate offspring (using the GAPF energy potentials);
    Parental replacement with phenotypic crowding;

**Algorithm 6:** Pseudocode of MaOEADRA Algorithm [Zou et al., 2021].

**Input**: Population size $N$, objectives $m$, the termination criterion;
**Output**: The final population $P$;

$W \leftarrow$ UniformReferencePoint($N$);
$P_0 \leftarrow$ RandomInitialize($N$);
$A \leftarrow P_0, V \leftarrow W$;
$R \leftarrow Zeros_{N\times 1}$;
$Gen \leftarrow 1$;
**while** *the termination criterion is not satisfied* **do**
    $O \leftarrow Variation(MatingSelection(P_t, N))$;
    $Qt \leftarrow P_t \cup O$;
    $[A, V, R] \leftarrow$ UpdateRefPoint($A \cup O, W, V, R, Gen$);
    $P_{t+1} =$ EnvironmentalSelection($Q_t, V, A$);
    $Gen \leftarrow Gen + 1$;
Return $P$;

**Algorithm 7:** Pseudocode of IDEA Algorithm [Xia et al., 2023].

**Input:** Population size $N$, objectives $M$, the termination criterion;
**Output:** Final population;

Generate uniformly distributed reference points $RP$ and an initial population $P_0$;
**while** *the termination criterion is not satisfied* **do**
    Calculate $I^r_\infty(p_x|p_y)$ between any two solutions and save the result to matrix $I_M$;
    Find neighbor solutions into $S_n$ by using $I_M$;
    Crossover and mutation to generate offspring $Q_t$ by using $S_n$;
    $P_{(t+1)} =$ EnvironmentalSelection($P_t \cup Q_t, I_M, RP$);
    **if** *the problem is irregular* **then**
        $RP =$ LearningPopulation($P_{(t+1)}, N$);
    $t = t + 1$;
Return $P_t$

# References


[Deb and Jain, 2014] Deb, K. and Jain, H. (2014). An evolutionary many-objective optimization algorithm using reference-point-based nondominated sorting approach, Part I: Solving problems with box constraints. *IEEE Transactions on Evolutionary Computation*, 18(4):577–601.

[Li et al., 2015] Li, K., Deb, K., Zhang, Q., and Kwong, S. (2015). An evolutionary many-objective optimization algorithm based on dominance and decomposition. *IEEE Transactions on Evolutionary Computation*, 19(5):694–716.

[Liu et al., 2019] Liu, J., Wang, Y., Wang, X., Guo, S., and Sui, X. (2019). A new dominance method based on expanding dominated area for many-objective optimization. *International Journal of*

*Pattern Recognition and Artificial Intelligence*, 33(03):1959008.

[Praditwong and Yao, 2006] Praditwong, K. and Yao, X. (2006). A new multi-objective evolutionary optimisation algorithm: The two-archive algorithm. In *2006 International Conference on Computational Intelligence and Security*, volume 1, pages 286–291.

[Rocha et al., 2017] Rocha, G. K., Angelo, J. S., Santos, K. B., Custódio, F. L., Dardenne, L. E., and Barbosa, H. J. (2017). Using an aggregation tree to arrange energy function terms for protein structure prediction. In *2017 IEEE Conference on Computational Intelligence in Bioinformatics and Computational Biology (CIBCB)*, pages 1–7.

[Xia et al., 2023] Xia, Y., Huang, J., Li, X., Liu, Y., Zheng, J., and Zou, J. (2023). A many-objective evolutionary algorithm based on indicator and decomposition. *Mathematics*, 11(2):413.

[Zou et al., 2021] Zou, J., Zhang, Z., Zheng, J., and Yang, S. (2021). A many-objective evolutionary algorithm based on dominance and decomposition with reference point adaptation. *Knowledge-Based Systems*, 231:107392.
